# Influence of *Helicobacter pylori* culture supernatant on the ecological balance of a dual-species oral biofilm

**DOI:** 10.1590/1678-7757-2017-0113

**Published:** 2018-02-15

**Authors:** Wenling Zhang, Xiaohong Deng, Xuedong Zhou, Yuqing Hao, Yuqing Li

**Affiliations:** 1 Sichuan University Sichuan University West China Hospital of Stomatology State Key Laboratory of Oral Diseases, National Clinical Research Center for Oral Diseases Chengdu People's Republic of China State Key Laboratory of Oral Diseases, National Clinical Research Center for Oral Diseases, West China Hospital of Stomatology, Sichuan University, Chengdu, People's Republic of China.

**Keywords:** *Helicobacter pylori*, *Streptococcus mutans*, *Streptococcus sanguinis*, Oral biofilm, Ecological balance

## Abstract

Dental caries is a chronic progressive disease occurring in the tooth hard tissue due to multiple factors, in which bacteria are the initial cause. Both *Streptococcus mutans* and *Streptococcus sanguinis* are main members of oral biofilm. *Helicobacter pylori* may also be detected in dental plaque, playing an important role in the development of dental caries.

**Objective:**

The aim of this study was to investigate the effect of *H. pylori* culture supernatant on *S. mutans* and *S. sanguinis* dual-species biofilm and to evaluate its potential ability on affecting dental health.

**Material and methods:**

The effect of *H. pylori* supernatant on single-species and dual-species biofilm was measured by colony forming units counting and fluorescence *in situ* hybridization (FISH) assay, respectively. The effect of *H. pylori* supernatant on *S. mutans* and *S. sanguinis* extracellular polysaccharides (EPS) production was measured by both confocal laser scanning microscopy observation and anthrone-sulfuric acid method. The effect of *H. pylori* supernatant on *S. mutans* gene expression was measured by quantitative real-time PCR (qRT-PCR) assays.

**Results:**

*H. pylori* supernatant could inhibit both *S. mutans* and *S. sanguinis* biofilm formation and EPS production. *S. sanguinis* inhibition rate was significantly higher than that of *S. mutans*. Finally, *S. mutans* bacteriocin and acidogenicity related genes expression were affected by *H. pylori* culture supernatant.

**Conclusion:**

Our results showed that *H. pylori* could destroy the balance between *S. mutans* and *S. sanguinis* in oral biofilm, creating an advantageous environment for *S. mutans*, which became the dominant bacteria, promoting the formation and development of dental caries.

## Introduction

*Helicobacter pylori* is implicated in several diseases such as gastritis, gastric ulcers and gastric carcinoma^20^^,^^26^. Approximately 10% of individuals suffer from gastritis or gastric ulcer due to *H. pylori* infection^28^. *H. pylori* can also be detected in saliva, on the dorsum of the tongue, on the surface of oral ulceration and in dental plaque^8^^,^^15^^,^^22^, the latter representing a crucial location, playing an important role in the development of dental caries. The prevalence of *H. pylori* infection in the oral cavity of gastric *H. pylori*-positive people is significantly higher than that of gastric *H. pylori-*negative people^30^. Researchers increasingly consider *H. pylori* as a conditional pathogen that exists in the oral cavity of both healthy people and patients with gastritis^14^. *H. pylori* infection in the oral cavity is associated with dental caries and poor oral hygiene. The caries rate in *H. pylori*-positive people is higher than that in *H. pylori*-negative people^14^.

According to the World Health Organization (WHO), dental caries has been one of the most important global oral health issues, accounting for 60-90% school-aged children of most of the industrialized countries^27^. *Streptococcus mutans* is considered a crucial agent in caries pathogenesis because of its cariogenic traits^9^^,^^23^. Glucans are essential to the adhesion of *S. mutans* to the tooth surface and to other oral bacteria, as well as to the formation of dental biofilms matrix^11^^,^^29^. Furthermore, *S. mutans* possesses aciduric properties, allowing it to perform glycolysis at low pH values within the matrix of the biofilm, which result in dental enamel demineralization^24^. *Streptococcus sanguinis* is usually colonizing oral biofilm^12^, having been considered a “good” member in the oral biofilm, since its presence is associated with the absence of caries^13^. *S. mutans* and *S. sanguinis* inversely affect each other in the formation of dental plaque^13^^,^^21^. Previous studies have shown that the interspecies interaction between *S. mutans* and *S. sanguinis* is mediated by *S. mutans* acidogenicity (production of lactic acid by L-lactate dehydrogenase, encoded by *ldh*) and production of bacteriocin (two major mutacins, mutacin IV and mutacin V, encoded by *nlmAB* and *nlmC*, respectively)^13^. These two streptococci compete for teeth colonization, since elevated levels of *S. sanguinis* in the early colonization results in a delayed colonization by *S. mutans*. Conversely, *S. mutans* teeth colonization is associated with low levels of *S. sanguinis*^13^. Indeed, caries-free children have high levels of *S. sanguinis* in their saliva and dental plaque compared to children with carious lesions who, instead, showed an elevated concentration of *S. mutans*^7^. Therefore, the imbalanced microecology of dental plaque was considered a key factor leading to caries. Currently, several studies^10^^,^^14^^,^^15^^,^^25^ are available to demonstrate the relationship between oral *H. pylori* and dental caries, although the mechanism is still unclear. In this study we analyzed the effects of *H. pylori* culture supernatant on *S. mutans* and *S. sanguinis* dual-species biofilm formation.

## Material and methods

### Bacterial strains and growth conditions

*H. pylori* ATCC 43504 was incubated in brain heart infusion (BHI) fluid medium with 5% Fetal Bovine Serum (FBS) that represented the *H. pylori* medium, in a microaerophilic chamber (6% O_2_, 10% CO_2_, and 84% N_2_; Thermo Fisher Scientific, Inc., Waltham, MA, USA). *S. mutans* UA159 (ATCC 700610) and *S. sanguinis* (ATCC 10556) were maintained in BHI fluid medium in an anaerobic chamber (10% H_2_, 5% CO_2_, and 85% N_2_; Thermo Fisher Scientific, Inc., Waltham, MA, USA) for planktonic growth. Both *S. mutans* and *S. sanguinis* were grown in BHI with 1% (w/v^-1^) sucrose as a supplemental carbohydrate source, to allow biofilm formation. Biofilms were incubated at 37°C without agitation.

### *H. pylori* supernatant collection

*H. pylori* stored at -80°C was incubated in BHI fluid medium with 5% FBS in the mentioned microaerophilic environment to allow their recovery, and subsequently subcultured for four days. The bacterial solution was centrifuged at 4000 g · min^-1^ for 10 min; the supernatant was collected and subsequently filtered by a 0.22 μm filter under aseptic conditions. The supernatant, named *H. pylori* supernatant, was stored at -20°C until use.

### Planktonic growth assay

*S. mutans* and *S. sanguinis* overnight bacterial cultures were diluted to an OD_600nm_=0.2 (according to McFarland turbidity standards) in BHI and placed in a sterile 96-well microtiter plate to perform planktonic growth curve assay. Each well containing 190 μL *S. mutans* or *S. sanguinis* culture (OD_600nm_=0.2) plus 10 μL *H. pylori* supernatant represented the experimental group, while the control group was represented by the same 190 μL bacteria culture plus 10 μL *H. pylori* medium. Plates were incubated at 37°C and sampled at hourly intervals for 24 h. The optical density at 600 nm (OD600, transmittance) was recorded hourly using a microplate reader (BioTek, Winooski, VT, USA) as previously described^19^. Four replicates of each bacterium for each group were used.

### Fluorescence *in situ* hybridization (FISH)

Overnight bacterial cultures of S. *mutans* and S. *sanguinis* were adjusted to OD_600nm_=0.2 in fresh BHI supplemented with 1% sucrose. Cultures of each bacterial species were inoculated either sequentially at a 3 h interval or simultaneously on saliva-coated glass coverslips in a 24-well cell culture plate. The experimental groups contain 1.5 mL BHI with 1% (w/v^-1^) sucrose, 200 μL *S. mutans* culture, 200 μL *S. sanguinis* culture and 100 μL *H. pylori* supernatant, while the control groups had 100 μL *H. pylori* medium (BHI with 5% FBS) instead of *H. pylori* supernatant. Finally, standard 24-well cell culture plates were incubated at 37°C under anaerobic condition for 24 h.

After the 24-h incubation period, biofilms were fixed in 4% paraformaldehyde, labeled with oligonucleotide probes (Probe 5′-ACTCCAGACTTTCCTGAC-3′ specific for *S. mutans* was labeled with FITC and probe 5′-GCATACTATGGTTAAGCCACAGCC-3′ specific for *S. sanguinis* was labeled with ROX) and analyzed by species-specific FISH as previously described^3^. Micrographs from at least five randomly selected fields of each sample were captured. S. *mutans* to S. *sanguinis* ratio was calculated based on the coverage area of each species as determined by IMAGE PRO PLUS 6.0 analysis (Media Cybernetics, Silver Spring, MD, USA).

### Biofilm single colony forming units (CFU) count

Standard 24-well cell culture plates were filled with 200 μL *S. mutans* or *S. sanguinis* culture (OD_600nm_=0.2) and *H. pylori* supernatant in BHI to a final amount of 2 mL containing 1% (w/v^-1^) sucrose in total. The control group had the same composition except the 5% *H. pylori* medium that replaced the 5% *H. pylori* supernatant. Plates were incubated at 37°C under anaerobic conditions for 24 h. Plates were washed twice with sterile PBS to remove planktonic and loosely adherent cells. Adherent cells from the biofilm were resuspended by vigorous pipetting and vortexing and were serially diluted 10^6^-fold through 10^8^-fold and plated onto BHI agar plates. Each group was performed in triplicate. Finally, plates were incubated at 37°C under anaerobic conditions for 48 h. Colony forming units (CFU) were quantified to evaluate the *H. pylori* supernatant inhibition ratio.

### Confocal laser scanning microscopy

Sterilized coverslips (1 cm in diameter) were placed into each well of standard 24-well cell culture plates to allow biofilms development. The standard 24-well cell culture plates contained 200 μL *S. mutans* or *S. sanguinis* culture (OD_600nm_=0.2) and *H. pylori* supernatant in BHI to a final amount of 2 mL containing 1% (w/v^-1^) sucrose in total. Alexa Fluor 647 (10 000 MW; Molecular Probes, Invitrogen, Carlsbad, CA, USA) was added to each well to label the formed extracellular polysaccharides (EPS) as previously described^3^. The control group had the same composition except that 5% *H. pylori* supernatant was replaced by 5% *H. pylori* medium.

The plate was incubated at 37°C under anaerobic conditions for 24 h. Next, we removed the planktonic bacteria, washed the coverslips with sterile PBS and dried them with a sterile filter paper, keeping them in the dark for the entire procedure. Bacteria were stained with SYTO 9 (Molecular Probes, Invitrogen, Carlsbad, CA, USA) as previously reported^3^ and coverslips were washed using deionized sterile water to remove the residual dye, dried with a sterile filter paper, and sealed with immersion oil type-F for laser scanning confocal microscopy (Leica TCS SP2; Leica Microsystems, Wetzlar, Germany) provided with a 63× oil immersion objective lens. Image collection gates were set at 655 to 690 nm for Alexa Fluor 647 and at 495 to 515 nm for SYTO 9. During imaging, amplifier gain (1.0), detector gain (500 V), and offset (0%) were kept constant. Five randomly selected fields were scanned for each sample. The quantification of EPS/bacteria biomass was performed with IMARIS 7.0.0 (Bitplane, Zurich, Switzerland), as previously described^19^.

### Anthrone-sulfuric acid method to determine biofilm insoluble EPS production

Standard 24-well cell culture plates contained 200 μL *S. mutans* or *S. sanguinis* culture (OD_600nm_=0.2) and *H. pylori* supernatant in BHI to a final amount of 2 mL containing 1% (w/v^-1^) sucrose in total. The control group had the same composition except that 5% *H. pylori* supernatant was replaced by 5% *H. pylori* medium. The plate was incubated at 37°C under anaerobic conditions for 24 h. Next, planktonic bacteria were removed and the adherent biofilm was resuspended in 2 mL PBS. The detailed procedure was performed as previously described^19^. Water-insoluble extracellular polysaccharides were extracted from the sample using 1.0 M NaOH with agitation for 2 h at 37°C^19^. The concentration of alkali-soluble carbohydrate was determined in the supernatant using the anthrone-sulfuric method. Briefly, the alkali-soluble carbohydrate solution was mixed with three volumes of anthrone-sulfuric acid reagent and heated in a water bath at 95°C for 5 min until the reaction was complete^19^. Then, the solution was allowed to cool-down to room temperature, and its absorbance was measured in a 96-well cell culture plate at 625 nm using a microplate reader (BioTek).

### Quantitative real-time PCR (qRT-PCR)

**Figure 1:**
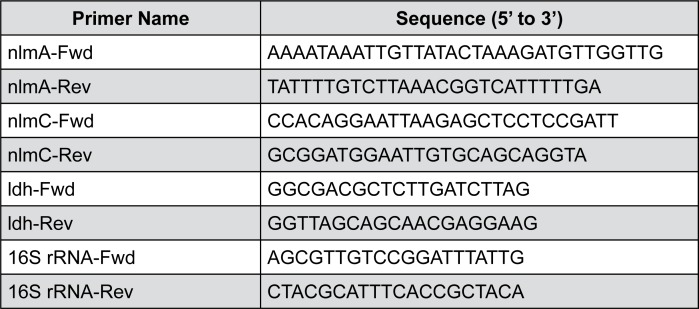
Primers used for qRT-PCR assays in this study

Gene-specific primers for *nlmA*, *nlmC*, and *ldh* were designed, as shown in Figure 1. Total bacterial RNA was isolated, purified, cDNA was reverse transcribed, and PCR reactions were performed as previously described^6^. Different gene expressions were normalized to 16S rRNA gene levels. Data were analyzed according to the 2^-ΔΔCT^ method^3^.

### Data analysis and statistics

In this study, all of the *in vitro* experiments include biological and technical triplicates. Exploratory data analysis was performed to determine the most appropriate statistical tests. Assumptions of equal variances and normal distribution of errors were also checked. Data were further analyzed using SPSS 16.0 (SPSS, Inc, Chicago, IL, USA), and unpaired Student's *t*-test was used to compare data of two groups. Results are calculated as average values ±SD (standard deviation). Data were considered significantly different if the two-tailed *P*-value was <0.05.

## Results

### Effect of *H. pylori* culture supernatant on *S. mutans* or *S. sanguinis* single-species biofilm formation

**Figure 2:**
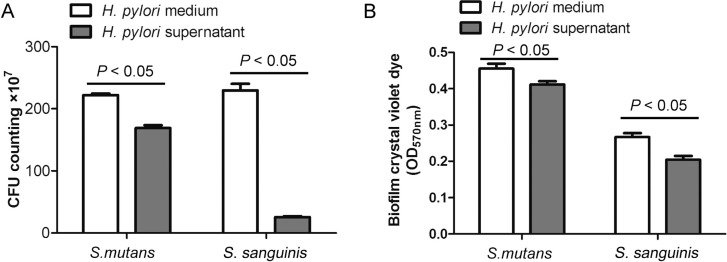
Effect of *H. pylori* supernatant on *S. mutans* and *S. sanguinis* biofilm formation. Planktonic bacteria were removed from *S. mutans* or *S. sanguinis* culture and the adherent biofilm was resuspended and diluted. A quantity of 100 μL of the final bacterial solution was placed onto the BHI agar plate. Colony forming units (CFU) were quantified to evaluate *H. pylori* supernatant inhibition ratio. Each group was performed in triplicate. Data were considered significantly different if the two-tailed P-value was <0.05. (A) Effect of *H. pylori* supernatant and *H. pylori* medium on single-species biofilm measured by CFU counting. (B) Effect of *H. pylori* supernatant and *H. pylori* medium on single-species biofilm measured by crystal violet dye staining

In the single-species biofilm formation, *S. mutans* and *S. sanguinis* CFU levels showed a statistically significant reduction due to the presence of *H. pylori* supernatant and not to *H. pylori* medium. Indeed, *S. mutans* CFU levels were 222×10^7^ CFU/mL and 169×10^7^ CFU/mL in the presence of *H. pylori* medium and *H. pylori* supernatant respectively, whereas *S. sanguinis* CFU levels were 230×10^7^ CFU/mL and 25×10^7^ CFU/mL, respectively. The inhibition rate exerted on *S. sanguinis* by *H. pylori* supernatant was statistically significantly higher than that exerted on *S. mutans* (Figure 2A) (F_(3,20)_=1.576; R^2^=0.9935; *P*<0.05). The inhibition of *S. sanguinis* and *S. mutans* by *H. pylori* supernatant was also confirmed by crystal violet dye staining of single-species biofilm (Figure 2B) (F_(3,20)_=0.9551; R^2^=0.9779; *P*<0.05).

### Effect of *H. pylori* culture supernatant on dual-species biofilm

**Figure 3:**
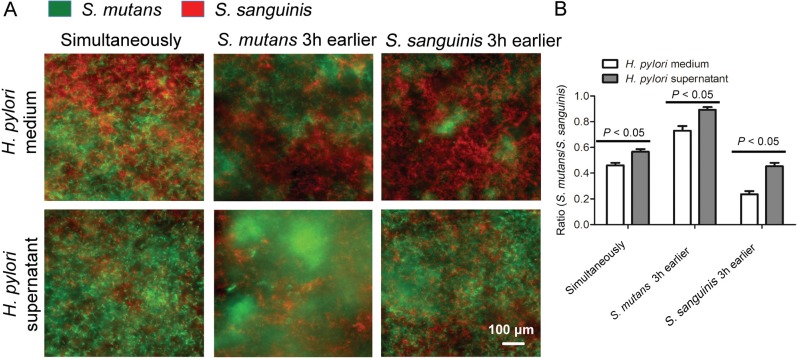
Effect of *H. pylori* supernatant on dual-species oral biofilm. Overnight bacterial cultures of *S. mutans* and *S. sanguinis* were inoculated either sequentially at a 3 h interval, or simultaneously on saliva-coated glass cover slips. After 24 h incubation, biofilms were fixed, labeled and analyzed by species-specific FISH assays as described in “Material and methods”. Data were considered significantly different if the two-tailed P-value was <0.05. (A) FISH images of dual-species biofilm were taken by confocal laser scanning microscopy (60× magnification). Green: *S. mutans*; Red: *S. sanguinis*. (B) *S. mutans* to *S. sanguinis* ratio in dual-species biofilm. Results were averaged from five randomly selected fields of each sample and are expressed as mean ±standard deviation

Since *H. pylori* could be detected in dental plaque and is related to the presence of dental caries, we examined the effect of *H. pylori* culture supernatant on the ecological balance of a dual-species biofilm composed by *S. mutans* and *S. sanguinis*. The 24-h dual-species bacteria biofilm formation results showed that the *S. mutans*/*S. sanguinis* ratio in the experimental groups treated with *H. pylori* supernatant was higher than that in the control group treated with *H. pylori* medium (Figures 3A and 3B) (F_(5,30)_=0.5442; R^2^=0.9959; *P*<0.05). The *S. mutans/S. sanguinis* ratio was highest in the group in which *S. mutans* was incubated 3 h earlier than *S. sanguinis*, while the lowest ratio was in the group in which *S. sanguinis* was incubated 3 h earlier than *S. mutans* (F_(5,30)_=0.5442; R_2_=0.9959; *P*<0.05). In other words, the microbial colonization was dominated by *S. mutans* in the groups treated with *H. pylori* supernatant.

### Effect of *H. pylori* culture supernatant on *S. mutans* or *S. sanguinis* EPS production

**Figure 4:**
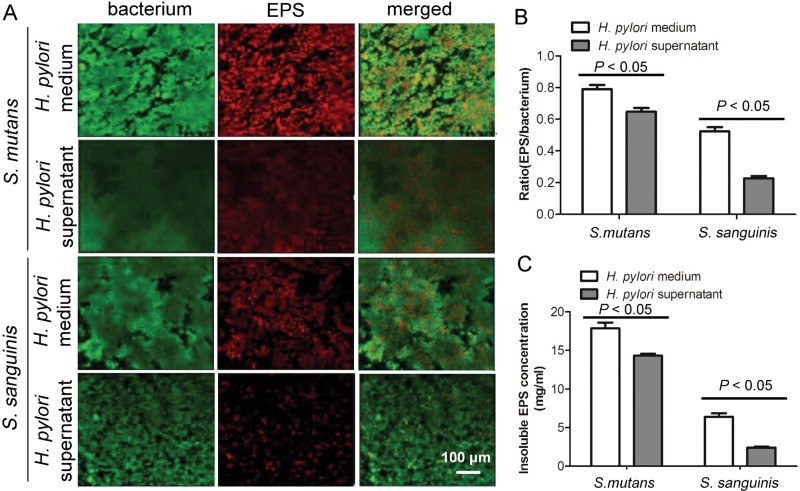
Effect of *H. pylori* supernatant on *S. mutans* and *S. sanguinis* biofilm and EPS. The effects of *H. pylori* supernatant on *S. mutans* and *S. sanguinis* biofilm and EPS were measured by both confocal laser scanning microscopy observation and anthranone sulfuric acid method as described in “Material and methods”. Data were considered significantly different if the two-tailed P-value was <0.05. (A) Confocal laser scanning microscopy images of single-species biofilms microscopy (60× magnifications). Live bacteria are green, EPS are red. (B) EPS to bacterium ratio in single-species biofilm. Results were averaged from three randomly selected fields of each sample and are expressed as mean ±standard deviation. (C) Anthrone-sulfuric acid method to determine the biofilm insoluble EPS production treated with *H. pylori* supernatant and *H. pylori* medium

We found, as expected, a decrease in both the biofilm and the EPS after *H. pylori* supernatant treatment if compared with the biofilm after *H. pylori* medium treatment. In addition, *H. pylori* supernatant inhibited EPS production in both bacteria, although the inhibiting effect on *S. sanguinis* was statistically significantly stronger than that on *S. mutans* (Figures 4A and 4B) (*P*<0.05). The Results of the anthrone-sulfuric acid method to determine the biofilm insoluble EPS showed that *H. pylori* supernatant could inhibit the production of biofilm insoluble EPS in both bacteria, and also in this experiment we found that the inhibition rate on *S. sanguinis* was statistically significantly higher than that on *S. mutans* (Figure 3C) (F_(3,20)_=1.968; R^2^=0.9985; *P*<0.05). The results of anthrone-sulfuric acid method and confocal laser scanning microscopy techniques were consistent. Both experiments showed that *H. pylori* supernatant could inhibit the production of EPS in *S. mutans* and *S. sanguinis* biofilm.

### Effect of *H. pylori* culture supernatant on *S. mutans* bacteriocin- and acidogenicity-related genes expression

**Figure 5:**
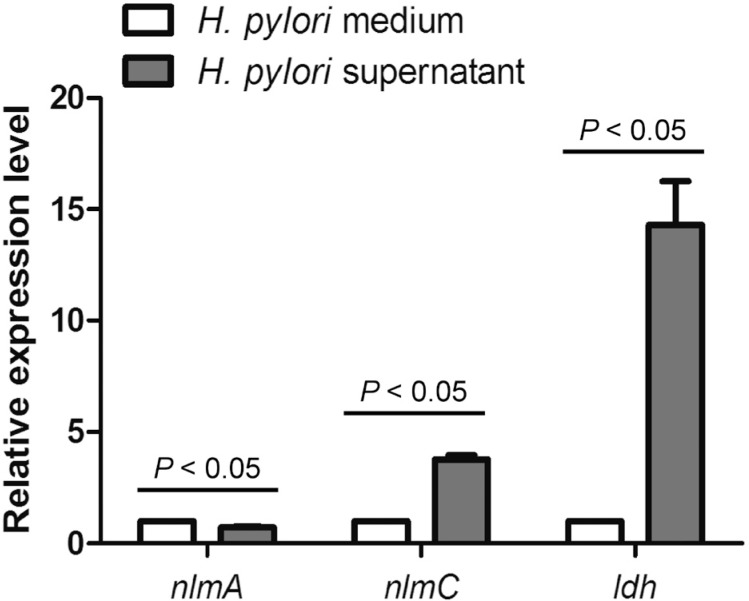
Effect of *H. pylori* supernatant on bacteriocin- and acidogenicity-related genes expression of *S. mutans*. qRTPCR assay was carried out as described in “Material and methods”. All genes were amplified using specific primers. Different gene expressions were normalized to 16S rRNA gene levels. Representative data are shown. Data were considered significantly different if the two-tailed P-value was <0.05

Previous studies have shown that interspecies interaction between *S. mutans* and *S. sanguinis* is mediated by *S. mutans* acidogenicity and production of bacteriocin^13^. *H. pylori* supernatant was able to increase the percentage of *S. mutans* in a dual-species biofilm. Therefore, to validate whether *S. mutans* bacteriocin- and acidogenicity-related gene expression was induced by *H. pylori* supernatant, we further investigated the expression of some *S. mutans* genes such as *nlmA*, encoding mutacin IV, *nlmC,* encoding mutacin V, and *ldh*, encoding L-lactate dehydrogenase (Figure 5). Although the expression of *nlmA* showed 0.7-fold change after the *H. pylori* supernatant treatment, *ldh* expression had a 3.8-fold increase, and *nlmC* expression had a 14.3-fold increase (*P*<0.05).

## Discussion

*H. pylori* is the first bacterium identified as a potential human carcinogenic pathogen^18^^,^^26^. It occurs in childhood by oral ingestion and persists for a lifetime in the host unless treated^20^. Several studies have demonstrated that *H. pylori* can be detected in dental plaque and saliva, making the oral cavity as the primary extra-gastric reservoir, which may be the source of infection and transmission^4^^,^^10^^,^^30^. The oral cavity is the starting point of the digestive tract, thus, because of the tight connection between oral cavity and digestive tract, the relationship between oral *H. pylori* and oral cavity diseases has caught increasing attention^5^. The association between *H. pylori* infection, dental caries and recurrent aphthous mouth ulcerations has been investigated in other researches^6^^,^^25^. A previous study showed that in *H. pylori* positive participants, caries prevalence rate was two times higher than in the participants without *H. pylori*^14^. Previous studies already have epidemiologic surveys regarding the relationship between dental caries and oral *H. pylori*, but the interaction between oral *H. pylori* and dental caries-related bacteria such as *S. mutans* and *S. sanguinis* has not been explored.

Oral biofilm can be defined as a diverse community of microorganisms, working as a system allowing bacterial adhesion and antibiotic resistance^16^. Oral biofilm is the key factor that causes dental caries, not bacterioplankton^2^^,^^17^. Thus, in this study we focused on oral biofilm to understand its role in caries formation. However, our attempts to culture *H. pylori* with other oral bacteria have failed because of the rigorous growth conditions needed by this bacterium^1^. Thus, in this study *H. pylori* supernatant was used to explore the influence of *H. pylori* on *S. mutans* and *S. sanguinis* biofilm. Our results showed that *H. pylori* supernatant could inhibit both *S. mutans* and *S. sanguinis* biofilm formation and EPS production. However, in a dual-species biofilm model, *S. mutans* showed a superior competitive advantage over *S. sanguinis* under *H. pylori* supernatant treatment. The observations in gene expression assays suggested that *H. pylori* supernatant could induce the production of mutacin and enhance the acidogenicity of *S. mutans*, alluding the creation of an advantageous environment for *S. mutans*, which became the dominant bacteria. We also found that the production of EPS of *S. mutans* and *S. sanguinis* was inhibited by *H. pylori* supernatant. Thus, we hypothesized that *H. pylori* supernatant contained some specific substances that may be secreted effectors, small molecules or metabolites, and that these substances could inhibit streptococcal EPS synthesis, affecting, therefore, the biofilm formation. However, this hypothesis needs further studies to be confirmed.

We also found that *H. pylori* supernatant had no significant effect on planktonic growth although having clear effects on biofilm and EPS formation. The biofilm formation and EPS production of *S. mutans* were known to be regulated by several signal transduction systems, like two-component system and second messenger signaling^3^. It is possible that the function of these signal systems was affected by substances in *H. pylori* supernatant. Our further research will focus on the specific mechanisms of the anti-biofilm effects of *H. pylori* supernatant.

In conclusion, our results showed the ability of *H. pylori* to destroy the balance between *S. mutans and S. sanguinis* in oral biofilm, creating an environment in which *S. mutans* is the dominant bacteria, promoting the formation and development of dental caries.
